# A Systematic Review of Electronic Medical Record Driven Quality Measurement and Feedback Systems

**DOI:** 10.3390/ijerph20010200

**Published:** 2022-12-23

**Authors:** Candice Donnelly, Anna Janssen, Shalini Vinod, Emily Stone, Paul Harnett, Tim Shaw

**Affiliations:** 1 Faculty of Medicine and Health, University of Sydney, Camperdown, NSW 2006, Australia; 2 Liverpool Cancer Therapy Centre, South Western Sydney Local Health District, Liverpool, NSW 2170, Australia; 3 South West Sydney Clinical Campuses, University of New South Wales, Liverpool, NSW 2170, Australia; 4 Department of Thoracic Medicine and Lung Transplantation, St Vincent’s Hospital, Darlinghurst, NSW 2010, Australia; 5 School of Clinical Medicine, University of New South Wales, Randwick, NSW 2031, Australia; 6 Crown Princess Mary Cancer Centre, Western Sydney Local Health District, Westmead, NSW 2145, Australia

**Keywords:** electronic medical records, quality improvement, digital health

## Abstract

Historically, quality measurement analyses utilize manual chart abstraction from data collected primarily for administrative purposes. These methods are resource-intensive, time-delayed, and often lack clinical relevance. Electronic Medical Records (EMRs) have increased data availability and opportunities for quality measurement. However, little is known about the effectiveness of Measurement Feedback Systems (MFSs) in utilizing EMR data. This study explores the effectiveness and characteristics of EMR-enabled MFSs in tertiary care. The search strategy guided by the PICO Framework was executed in four databases. Two reviewers screened abstracts and manuscripts. Data on effect and intervention characteristics were extracted using a tailored version of the Cochrane EPOC abstraction tool. Due to study heterogeneity, a narrative synthesis was conducted and reported according to PRISMA guidelines. A total of 14 unique MFS studies were extracted and synthesized, of which 12 had positive effects on outcomes. Findings indicate that quality measurement using EMR data is feasible in certain contexts and successful MFSs often incorporated electronic feedback methods, supported by clinical leadership and action planning. EMR-enabled MFSs have the potential to reduce the burden of data collection for quality measurement but further research is needed to evaluate EMR-enabled MFSs to translate and scale findings to broader implementation contexts.

## 1. Introduction

Quality measurement is essential to systematically identify unwarranted variation in care delivery. Over the last 20 years, measurement-feedback systems (MFSs) such as Audit and Feedback have been widely used in quality improvement programs to provide health professionals with information that reflects the care delivered. These MFSs are based on the theory that health professionals are prompted to improve care when the gap between current practice and optimal practice is highlighted [1,2]. Unlike clinical decision support tools used at the point-of-care, MFSs are a quality improvement tool to encourage health professionals and clinical teams to reflect on insights related to the quality of care delivery after the clinical episode has occurred [3]. MFSs often utilize quality indicators as objective measures of healthcare structures, processes, and outcomes [4], with the addition of benchmarks to provide standards of care. Internationally, healthcare systems collect and manage data to support the measurement of quality indicators, including government public reporting, cost analyses, safety audits, and college accreditation. Although these quality measurement activities are extensively deployed, variation exists in their documented utilization, and impact on clinical practice and patient outcomes [2,5]. Furthermore, these activities are often conducted at a population-based level and disconnected from clinical care delivery within hospitals.

There is a paucity of research on the specific aspects of MFSs that may influence their impact, reporting a wide range of factors associated with feedback utilization including data sources for analysis, feedback content and display, and implementation context [5,6,7]. Historically MFSs uses data sources such as large clinical registries and administrative databases, or manual chart abstraction [8,9]. Whilst there are benefits to the secondary use of registries and administrative databases, some challenges have been identified in their use in this context [10,11,12]. This may be attributed to the design of such data sources which were not intended for quality measurement and therefore may not contain variables needed to calculate relevant clinical process indicators [13]. Moreover, the collation of these data sources is highly resource-intensive and access is limited [14]. Therefore, resulting measurement and feedback are often significantly time-delayed, reducing clinical relevance and impact on care delivery [9].

The increasing quality reporting requirements expected of healthcare organizations have resulted in additional siloed data collection, duplication of effort, measurement burden, and increased expense [13,14,15]. These issues cast some doubt on existing methods for sourcing data to analyze the quality of care and present a need to explore more readily available data sources and methods to support technology-enabled MFSs. One potential data source that may overcome issues of clinical data relevance and temporality is the data routinely collected within Electronic Medical Records (EMRs). Since the development of the first EMR in 1972, EMR technology has significantly advanced. Particularly in the last decade, EMR packages have been developed and implemented in a variety of healthcare settings across the world [16,17,18,19]. With this widespread adoption of EMRs, the routine collection of comprehensive patient data continues to evolve. More recently there has been an increased interest in leveraging EMR data for secondary purposes including quality improvement [20,21,22]. Coupled with recent advances in technology enabling more efficient data extraction, manipulation, and feedback, updating traditional MFSs to utilize EMR data could increase access to timely, relevant, and actionable information. This would have a significant benefit for hospital efficiency, quality of care delivery and patient outcomes [23,24].

Despite this growing opportunity, little is known about the feasibility and effectiveness of EMR-enabled MFSs in tertiary care. Recent literature has explored electronic audit and feedback in primary care [25], theoretical concepts used in audit and electronic feedback [26], and dashboard interface features to support reflection on practice [27]. These studies included any data source, were limited to a small number of RCTs, or restricted to primary care. This study aims to extend current knowledge in secondary EMR use for quality improvement and explore the effectiveness and characteristics of published EMR-enabled MFSs in tertiary care settings. A systematic review was conducted on quality improvement interventions using EMR data as the primary source of quality measurement and feedback interventions for healthcare professionals and teams in tertiary care. The objectives of the review were to identify; (1) the effect of EMR-enabled MFSs on the quality of care and patient outcomes, and (2) the intervention characteristics of EMR-enabled MFSs.

## 2. Materials and Methods

### 2.1. Search Strategy

The search strategy was guided by the PICO Framework [28]. Studies were included in the review if they described an evaluation of the use and impact of EMR-enabled quality measurement and feedback. In the context of this review, EMR is used as a broad term for computerized data collection systems for collecting routine patient and treatment information, including terms such as; electronic health record (EHR), and electronic patient record (EPR). It is recognized that some EMR-enabled MFSs may have utilized data from other sources to complement EMR data as the primary data source and were included in this review, i.e., patient administrative systems. The search strategies listed in Supplementary File S1: Search Strategy were executed in four databases (MEDLINE, EMBASE, CINAHL, and the Cochrane Central Register of Controlled Trials) for the dates 1 January 2009–11 January 2022. The databases were selected due to their common use in health services research. The contemporary time period was selected relevant to socio-environmental context of EMRs and adoption in clinical practice [16,29,30]. String terms were developed using MeSH and free-text terms referring to key concepts; (1) healthcare professionals; (2) measurement feedback; and (3) EMRs. The search was restricted to studies in English. A hand search of the reference lists of identified relevant papers and a citation search of relevant papers was also conducted.

### 2.2. Data Management

Citations retrieved from the search were imported into the reference manager software program EndNote X9 for de-duplication, then imported into Covidence Systematic Review software a web-based platform for screening.

### 2.3. Study Selection

Two authors (CD and ES) independently screened the titles and abstracts against the exclusion criteria in Supplementary File S1: Search Strategy. When uncertainty arose, complete manuscripts were sought and any disagreements were resolved through discussion. Full-text manuscripts were screened by one reviewer (CD), and justifications for inclusion or exclusion were confirmed by a second member of the research team (ES). Key criteria excluded studies which were conference proceedings, lacked an intervention, delivered feedback only to student clinicians, were implemented in primary care, focused on a clinical decision support tool delivered at the point-of-care, EMR data was used as a supplementary data source, study outcomes were user-testing, improved outcomes were financially incentivized, only one instance of feedback was provided, or feedback did not include any quality measurement. Given the small number of studies in reviews with meta-analyses, this review included all intervention designs to provide context in the complexity of study interpretation, including intervention characteristics, the implementation, and the population [31,32].

### 2.4. Quality Assessment

The methodological quality of included studies was conducted by one reviewer (CD). The Quality Appraisal for Diverse Studies (QuADS) [33] tool was selected to conduct the appraisal of studies of multiple designs included in this review. The QuADS tool has demonstrated strong reliability for application in systematic reviews involving heterogenous studies of multi or mixed-methods in complex health services research [33]. The QuADS tool uses contains 13 reporting criteria scored on a scale from 0 to 3 (not at all/very slightly/moderately/complete). The QuADS tool advises against the use of a cut-off summary score of low-high quality, and therefore the quality appraisal is descriptively reported as the tool is intended.

### 2.5. Data Extraction

One author (CD) extracted data relevant to both intervention effect and design. A data abstraction template was developed in Microsoft Excel v. 16.34 to extract data. Guided by Cochrane’s EPOC extraction tool [34] the template included extraction of information related to the study methods and outcomes (i.e., author, country, year, study design, setting, duration, outcome measures). The capture of intervention characteristics was guided by data elements from previous audit and feedback reviews [2,26] including the aim of the intervention, unit of allocation for analysis and feedback, MFSs role in a wider quality improvement program, theoretical frameworks applied, content fed back, feedback presentation mode, interactive components, frequency of feedback, action planning used, peer comparison, and program sustainability strategies. As the focus of this review was on the specific use of EMR data, additional information regarding the data source(/s) for the MFS was collected. Author CD piloted the form on the first five articles, a second author (AJ) reviewed the form and minor refinements were made.

### 2.6. Data Synthesis and Analysis

This manuscript follows the PRISMA reporting guidelines where possible to discuss the synthesis of results [35]. This review included studies of different methodological designs and a meta-analysis was deemed inappropriate, therefore a narrative synthesis was performed. The reporting of study outcome metrics varied, however measures of intervention effect (direction of effect and *p* values) were synthesized where possible and otherwise outcomes were descriptively reported. The intervention characteristics were descriptively reported at the study level. Studies were grouped by key intervention characteristics, i.e., aim of intervention, feedback methods to explore any correlation. Tables were used to summarize study characteristics and reported outcomes, and intervention characteristics.

## 3. Results

**Figure 1:**
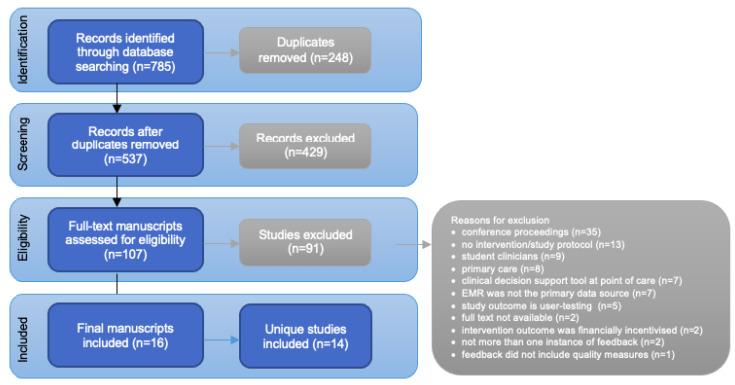
PRISMA flow-diagram shows the process of identifying records from database searches, screening abstracts and full-text manuscripts against the inclusion/exclusion criteria, and final included studies.

The literature search identified 785 records as demonstrated in Figure 1 [36]. After duplicates were removed, 537 potentially relevant abstracts were screened; where 429 were excluded. A total of 107 full-text manuscripts were retrieved for further screening, where 91 manuscripts were excluded based on the eligibility criteria. A final total of 16 manuscripts that discussed 14 unique EMR-enabled MFSs were included in the review. Two studies had multiple manuscripts associated with the reporting of intervention results.

### 3.1. Study Characteristics

A summary of key study characteristics are described in Table 1. The majority of studies used an uncontrolled before-and-after (BA) study design (n = 8), and the remaining were randomized controlled trials (RCTs) (n = 3) and interrupted time series (ITS) (n = 3) study designs. Using the QuADS tool in the appraisal of included studies; the quality of study design and reporting of criteria was highly variable. The QuADS tool identified areas of strengths and limitations in the included studies for consideration when interpreting results by applying a score of 0–3 for study elements of high-quality design. A clear quality concern was the lack of prospective comparative study designs which isolated the MFS impact on outcomes. Another weakness was the limited use of theoretical models/frameworks underpinning research. There was a large variation in QuADS tool scores for reporting sampling and participant sizes (range, n = 12–487), only two studies had >100 participants. All studies scored highly for an adequate description of study settings, noting location, types, and institution size. Studies were predominantly based in the United States (US) (n = 12). The two remaining studies were based in Sweden and Canada. The length of interventions varied among studies but most studies were ≥12 months in length (n = 10). There was a relatively even mix of single-center (n = 8) and multicenter studies (n = 6), ranging in institution size.

Almost all of the MFSs were part of a wider multifaceted quality improvement program (n = 12). Concurrent quality improvement strategies included new models of care, protocols and guidelines, education and training, clinical decision support tools, and EMR modifications, including implementation of pharmacy order sets. The study aims were well reported but highly varied. Both Hester et al., 2019 [37] and Dowling et al., 2020 [38] aimed to reduce low-value bronchiolitis management in pediatric care. Other similarities were found between studies that aimed to improve; pain management [39,40], prescribing practices [40,41,42,43], quality of discharge [41,42,44] and unnecessary test ordering [45,46]. The remaining studies had unique aims such as adherence to pneumonia guidelines [47], reducing heart failure re-admissions [48], improving lung-protective ventilation strategies [49], improving blood pressure control [50], and improving quality of glioma care [51,52]. As all studies utilized EMR data and routinely collected data sources in the intervention, data collection procedures and analytic methods were clear and detailed.

**Table 1:** Study Characteristics and Effect.

Study	Design	Setting	Population Size **	Outcome Measure(/s)	Effect Direction	Statistically Significant *
Banerjee et al. (2017) [48]	ITS	Single-center	Not Reported (NR)	re-admission	⇑	yes
				identifying heart failure patients	⇑	yes
Cline et al. (2016) [39]	BA	Multi-center (2 hospitals)	487	pain re-assessment	⇑	NR
Corson et al. (2015) [46]	BA	Multi-center (4 hospitals)	53	inappropriate test ordering	⇑	yes
				in-hospital mortality, blood transfusion	⇑	no
				LOS, re-admission	⇔	N/A
Dowling et al. (2022) [38]	ITS	Multi-center (7 hospitals)	47	bronchiolitis management	⇑	yes
				LOS	⇑	yes
				ICU admission, 72-hr ED revisit	⇔	N/A
Hester et al. (2019) [37]	BA	Single-center	20	bronchiolitis management	⇑	NR
				ED discharge, LOS, 7-day ED revisit	⇑	yes
				hospital admission LOS, readmission	⇑	no
Kestenbaum et al. (2019) [40]	BA	Single-center	NR	pain management	⇑	NR
				prescription costs	⇑	NR
Larkin et al. (2021) [45]	RCT	Multi-center (4 hospitals)	25	CT ordering	⇓	no
Navar-Boggan et al. (2014) [50]	BA	Single-center	42	blood pressure control	⇔	N/A
				repeat BP measurements	⇑	yes
Parks et al. (2021) [49]	ITS	Single-center	63	intra-operative lung-protective ventilation	⇑	yes
Patel et al. (2018) [44]	CRCT	Single-center	20 teams (n = NR)	discharge quality	⇑	yes
				30 day re-admission	⇑	no
Phase 1: Riblet et al. (2014) [52]	BA	Single-center	NR	peri-operative glioma care	⇑	yes
Phase 2: Riblet et al. (2016) [51]					⇑	no
Trent et al. (2019) [47]	CRCT	Single-center	16	sepsis/pneumonia management	⇑	yes
Phase 1: Stevens et al. (2017) [53]	BA	Multi-center (4 centers)	12	prescription of potentially inappropriate medications	⇑	yes
Phase 2: Vaughan et al. (2021) [42]		Multi-center (3 centers)	283		⇑	no
Wang et al. (2021) [43]	BA	Multi-center (5 centers)	18	opioid prescribing practices	⇑	NR
				opioids prescribed/month	⇑	yes
				opioids/prescription	⇑	no

* (*p* < 0.05, 95% CI); ** Number of health professionals receiving the intervention; Study design: RCT = randomized controlled trial, CRCT = cluster RCT, BA = before and after study, ITS = Interrupted time series; Effect direction: ⇑ = positive impact, ⇓ = negative impact, ⇔ = no change/mixed effect/conflicting findings.

### 3.2. Effect of MFSs on Quality of Care and Patient Outcomes

Details on outcomes, effect direction, and statistical significance * (*p* < 0.05, 95% CI) for each study are reported in Table 1. There is significant variability in how success was measured across interventions. Most studies measured the effect of the intervention in changes to the specific quality of care indicators targeted, whilst limited studies included the effect of the MFS on patient outcomes. Only one study had a primary measure of a patient outcome [50], three included secondary measures of multiple patient outcomes [37,38,46], and one had a single secondary patient outcome [44].

The majority of studies reported a positive effect on the primary outcome (n = 12), of which nine provided statistically significant results. Of the nine studies with statistically significant improvement, all had <70 participants, five were single-center and four were multi-center studies. All three ITS studies, and two CRCTs showed statistically significant improvement. In the two studies reporting null or negative effect, one reported no effect on blood pressure control [50] and the other reported a negative effect, where computed tomography (CT) orders increased significantly in both the intervention control groups and there was no significant difference between groups [45]. Nine of the studies reported secondary outcomes, of which most reported a positive effect (n = 8). With regard to the four studies that reported secondary outcomes related to the patients, two had positive effects, two had mixed effects (either positive or no effect).

### 3.3. MFS Characteristics

Key intervention characteristics were summarized in Table 2, and grouped by stages of an MFS; (1) data source and measurement, (2) feedback methods, and (3) facilitating action.

#### 3.3.1. Data Source and Measurement

In accordance with the inclusion criteria, all studies utilized EMR data as the primary source for analysis, however, additional data sources were used in four MFSs, including national registry data (n = 1), existing databases used in previous QI projects (n = 2), and patient satisfaction data (n = 1). All MFSs conducted quality measurements, mostly using quality indicators. Some MFSs used data to analyze a single quality indicator, guideline or behavior (n = 4), whereas others measured multiple quality indicators in a clinical focus area (n = 10). There was a relatively even mix of MFSs that measured care and outcomes on an individual provider level only (n = 7) and team or department level only (n = 3), and those that measured at both the individual and team, department or hospital level (n = 4).

#### 3.3.2. Feedback Methods

Across the 14 studies, the most common feedback recipients were ED clinicians (n = 3) and pediatric ED clinicians (n = 2). Some MFSs delivered feedback to specialty clinicians (n = 4) including cardiologists, palliative care clinicians, anesthetists, rheumatologists, and specialty teams (n = 3) including internal medicine teams, a cardiology multidisciplinary team (MDT) and neuro-oncology MDTs. Other feedback recipients included hospitalists (n = 1) and nurses (n = 1) across different hospital departments. The content of feedback was typically presented as quality indicators including rates of adherence with best-practice or trend data over time. Many MFSs used peer comparison with individuals or with other teams/hospitals (n = 9), as well as benchmarks of a regional or national standard. The majority of MFSs used reports to deliver feedback (n = 8). These reports were either emailed (n = 5) or hand-delivered (n = 2), one study did not specify. The remaining studies used electronic dashboards to provide a more visual and interactive feedback solution (n = 7). The interventions that used emailed reports displayed static data, whereas dashboard interventions updated and displayed the measurement data in near real-time (<24 h). Feedback reports were delivered in either quarterly (n = 1), monthly (n = 6), or weekly (n = 1) intervals, and dashboards were accessible throughout the intervention period.

#### 3.3.3. Facilitating Action

Six studies utilized a theoretical framework/model used to guide the design of the MFSs including the Plan-Do-Study-Act (PDSA) cycle (n = 2) [43,52], Vision-Analysis-Team-Aim-Map-Measure-Change-Sustain model and PDSA (n = 1) [41,42], Feedback Intervention Theory (n = 1) [39], Calgary Audit and Feedback Framework (n = 1) [38], and one study developed their own program theory [44]. Action planning, academic detailing, or coaching was used when providing feedback in five MFSs. Two dashboard studies included weekly and quarterly team reviews. These sessions were typically guided by a senior clinical leader or nominated process owner, statistician or research support.

## 4. Discussion

This systematic review identified 16 articles describing the results of 14 EMR-enabled MFSs delivered to healthcare professionals and teams within hospitals. The primary objective of this review was to identify the effect EMR-enabled MFSs have on the quality of care and patient outcomes. Overall, 12 of the 14 MFSs (86%) demonstrated a positive effect on various outcome measures. Although, as almost all studies implemented an MFS within a multifaceted quality improvement program, contamination exists in the measured effects. Three studies, however, did use interrupted time series studies [49] and were able to assess the MFS as an individual intervention strategy and identified significant improvements specific to the MFS phase. Another consideration is the heterogeneity in the study designs included in this review. Given that eight (53%) were uncontrolled before-and-after studies and therefore were not randomized, causal inferences and generalizability is limited. Due to this lack of high quality evidence available it is difficult to determine the definitive impact of using EMR data to drive MFSs. Despite this, all studies feasibly operationalized EMR data for the purpose of an MFS. Future study designs could benefit from a comprehensive description of the implementation context and isolating the evaluation of MFS in wider quality improvement studies. Furthermore, identified characteristics of MFSs and insights of EMR data utilized for this purpose may provide guidance in the development of future EMR-enabled MFSs.

Common characteristics which supported the included MFSs pertains to measurement of quality indicators at individual and team levels, the use of technology and tools in feedback (i.e., interactive dashboards), benchmarking (peer comparison, standards), and facilitating action with leadership (clinical champions, process owners) and active clinical engagement (goal setting and action planning). These characteristics of EMR-enabled MFSs are aligned with those found in previous reviews of characteristics of audit and feedback. Although studies in this review were not included in Tuti et al.’s [26] review of audit and electronic feedback using behavior change theory, the finding of limited use of theoretical frameworks to guide EMR-enabled MFSs was consistent with Tuti et al.’s review. Theoretical frameworks such as Payne and Hysong’s [7] model depicting aspects of audit and feedback that impact acceptance feedback could be considered in the design of future EMR-enabled MFSs, particularly where the EMR data source may influence the feedback content, timeliness, personalization, and trust in data. Van den Bulck et al.’s [25] review of electronic audit and feedback was limited to primary care and despite distinct differences between EMRs used in primary care clinics and more widely implemented hospital EMRs, similar levels of effectiveness were reported, extending the findings of EMR-enabled MFSs in the tertiary care context.

Whilst the findings discussed in this manuscript provide a broader range of feedback methods to the dashboard focus of Bucalon et al.’s [27] review, this review found all seven of the EMR automated dashboards to be effective. The included studies that used EMR-enabled dashboards to deliver feedback were published in the last five years, demonstrating the emergence of clinical analytics in healthcare and the literature. A widely reported benefit of utilizing dashboards in feedback was the timely access to the EMR data. The Stanford Heart Failure dashboard study [48] found the availability of real-time patient outcome measures for clinicians increased relevance to clinical workflow and contributed to program sustainability. The interactive feature of dashboards and the ability to drill down to specific cohorts or individual patient level supported use of the quality measurement data to identify areas or specific medical record numbers for further investigation. All dashboard studies discussed the multi-disciplinary design of dashboards, including clinical staff, business analytics, and IT. These multi-disciplinary groups met frequently, with some studies reporting weekly planning meetings. The involvement of health professionals as end-users was reported to increase dashboard usability and Cline et al. [39] noted that informal leaders emerged through a co-design process.

In addition to the design of the MFSs, many of the included studies actively engaged health professionals and clinical teams in both measurement and feedback components using formal leadership roles and clinical champions. All MFSs that used team review meetings and action planning in conjunction with feedback had positive outcomes. In Patel’s study [44] that included 15 min sessions of in-person intensive feedback, the MFS with action planning had statistically significant improvement but became non-significant when action planning ceased. One study appointed process owners for each quality measure, who acted as leaders and held responsibility for the quality improvement area and was found to be a significant contributor to sustained project success [52]. Studies reported that credible clinical leadership that encouraged the identification of clinical performance improvement opportunities reduced the stigma of MFSs as a punitive tool for lack of performance, and team collaboration created a sense of camaraderie, motivating the team to remain engaged in the project goals. EMR-enabled MFSs that used feedback with identified or de-identified peer comparison reported benchmarking influenced health professional behavior. Identified examples of this influence included regular non-judgmental conversation within units about quality measurement data and friendly competition amongst peers. Despite all studies focusing on a specific aspect of hospital care, no studies discussed the potential adverse effects of concentrating quality measurement and improvement efforts in a single area, which may include measurement fixation behavior, or quality improvement in one area occurring at the expense of quality of care in another [54].

This review focused on EMR data for quality improvement contributes to the growing literature on the secondary use of routinely collected data. A key finding from this review was an articulation of the challenges that need to be overcome when using EMR data for quality measurement and improvement. All studies in this review highlighted that MFS utilizing EMR data requires both technical knowledge and skills to extract data and a clinical understanding of decision-making, clinical pathways, and processes to manipulate data appropriately. Such efforts are dependent on the project timelines, IT capacity, or ability to collaborate with the EMR vendor to access proprietary databases. This is a commonly reported issue across secondary use of EMR data more broadly [55]. These challenges include accessing data that was recorded predominantly in clinical notes, rather than standardized structured EMR fields, making it difficult to translate into readily analyzable data for measuring the quality of care. Some studies identified these issues in the early stages of the quality improvement projects and modified EMR data fields to enhance data collection for MFSs by establishing a working relationship with EMR vendors.

Two EMR vendors, Epic System Corporation and Cerner Corporation were reported across the six studies which specified the specific EMR package. These two vendors hold a share of over 55% of the market in the US where the majority of studies were conducted [56,57]. This finding is consistent with reviews of EMR adoption, commonly reporting the majority of literature are based in the US [29,58], often linked to the implementation of the Health Information Technology for Economic and Clinical Health Act in 2009 to support the meaningful use of EMRs. This legislation provided a foundation for EMR quality improvement programs and therefore the studies included in this review may have more mature EMR systems, technology support, established workflows, and organisational culture supportive of data capture and use, and therefore more likely to participate in EMR-enabled MFSs [59].

Although EMR data was the primary source for analysis in all studies, additional data sources were utilized in five MFSs. Not all data required for measurement calculation existed in a single database, and therefore access to multiple databases was required to support MFSs. This suggests that EMR data alone may not capture sufficient data required for quality measurement and improvement. This review found clinical registries were the most commonly used data source to supplement EMR data. Clinical registries may provide access to additional longitudinal information such as death data, pertinent to the measurement of quality survival and mortality quality indicators. The automated extraction of EMR data into clinical registries has been explored in the literature and whilst it found the integration to be viable, complex challenges remain in the lack of standardization, quality of EMR data, and data completeness [20,60,61].

A commonly reported issue of EMRs is a lack of interoperability between systems used in different services, making consolidation of data across organizations difficult [16]. Whereas clinical registries typically collate data across larger regions for comparative analyses. Interoperability issues may have contributed to the smaller number of studies utilizing EMR data in MFSs to date. However, more recent policy changes and ancillary technology offer promising solutions for the secure transfer and use of standardized EMR data using Fast Health Interoperability Resources (FHIR) [62,63,64,65]. The use of FHIR data models enables EMR data transfer for secondary purposes and provides a foundation for future EMR-enabled MFSs. Patient satisfaction data was another source used to complement the use of EMR data. EMRs have historically lacked the systematic collection of patient and carer experiences of care, quality of life, and symptoms [66]. However, modern EMRs have developed and implemented patient-reported outcome modules which have the potential to improve patient-centered quality of care measurement [67].

In order for EMR data to further support MFSs and reduce the overall data collection burden, implementation of data standards across pertinent data elements should be carefully considered for meaningful secondary EMR data use in quality improvement. Some studies noted a lack of trust or time required to build trust in the data used within the MFS. A method used to build trust included engaging health professionals in the data from the outset of the project planning. One study incorporated initial team meetings using data for open discussion, to establish a common understanding of EMR documentation expectations, which data elements would be extracted for the MFS, and how the feedback reports or dashboard interfaces would be created. Studies that utilized these mechanisms reported trust as an enabler of clinical behavior change and fostered a non-judgmental culture of quality measurement.

### Limitations

Secondary use of EMR data is a rapidly developing area of research, and many studies may not yet be at the development stage of higher quality studies. The number of studies may also be limited by a lack of formal interventions in health service quality improvement activities or that there are a number of barriers to translating an MFS from proof-of-concept to a final product for evaluation. Therefore, a limitation of this review is the exclusion of earlier stage research such as conference proceedings, and studies that had an outcome of user testing. Including this research may be useful in understanding the development and application of EMR data for MFSs and increased the number of studies included in this review. Another limitation of this review is the restriction of the period of time (2009–2022). Although this contemporary time period was selected as relevant to implementation of modern EMRs and levels of adoption, publications pre-dating this period may have been missed. These limitations may provide additional insight into the lower number of studies. This review also excluded studies where medical students or trainee clinicians were the recipients of feedback. The large number of studies in this area may be attributed the learning context and culture of clinicians at this career level. The decision to exclude these studies was based on the difference in this context to ongoing professional development as a fully qualified clinician. Therefore, results may not be transferrable or comparable. Given a large number of studies in the student clinician context, there may be some learnings to glean from studies including students/trainee clinicians in a future review. Finally, this review excluded studies that utilized only registry data but it is important to note that in countries such as Sweden, Denmark, and the Netherlands, registries for certain clinical conditions have been integrated with automated EMR exports and have much shorter time delays than other clinical registries [9,68,69]. More advanced registry-based MFSs such as these were excluded from this review as it was difficult to determine the exact data sources that contributed to registries across all studies in the screening phase but such studies could be the focus of a future review.

## 5. Conclusions

EMRs contain rich information related to clinical care delivery that could be used in quality improvement programs. Overall, utilizing EMR data to drive MFSs has been demonstrated as feasible and is associated with some studies which show positive changes in care delivery, particularly in multicomponent quality improvement interventions. However, evidence of EMR-enabled MFS impact on patient outcomes is limited, highlighting the need for future high quality studies that would enable causal inferences to be drawn. Common characteristics of successful EMR-enabled MFSs, included additional data sources to supplement EMR data (clinical registries and patient-reported data), transparency in data use and quality measurement calculation, technology-enabled feedback methods (dashboards and emailed reports), and support of clinical leadership, goal setting and action planning to facilitate practice change. Our findings highlight the need to improve the quality and implementation of future studies designed to enable causal inferences to be drawn in secondary EMR data use for quality measurement and feedback.

## Figures and Tables

**Table 2:** MFS Characteristics.

Study	Goal	Data Source	Unit of Analysis	Content of Feedback	Feedback Delivery	Feedback Recipients	Action Facilitation	Co-Interventions
Banerjee et al. (2017) [48], United States	Reduce heart failure re-admissions	EMR (Epic Systems Corporation) + patient satisfaction data	Individual provider	Quality indicators (i.e., readmission rates for HF)	Interactive dashboard updated daily with drill-down options	Cardiology MDT	NR	New model of care
Cline et al. (2016) [39], United States	Improve adherence to pain management guidelines	EMR	Unit level	Quality indicators (i.e., rates of pain assessment)	Monthly emailed report	Nurses	Coaching, annual review	Education session
Corson et al. (2015) [46], Sweden	Reduce unnecessary test-ordering	EMR	Individual provider	A list of providers/no. of common labs ordered, case study examples	Monthly emailed report	Hospitalist providers	Academic detailing session	NR
Dowling et al. (2022) [38], Canada	Reduce low-value bronchiolitis management	EMR + national ambulatory care dataset	Individual provider (w/peer comparison)	Quality indicators (i.e., length of stay, ED revisits within 72 h)	Two data reports	Pediatric ED clinicians	Team feedback sessions, a commitment to change form (action planning)	NR
Hester et al. (2019) [37], United States	Reduce low-value bronchiolitis management	EMR (Cerner Corporation)	Individual with specific patient cohorts (w/peer comparison), and unit level	Quality indicators (i.e., use of chest radiographs, bronchodilators)	Interactive dashboard with drill-down options (voluntary dashboard use)	Pediatric ED clinicians	NR	Education and guideline disseminated prior to intervention, EMR order-set implemented 2 months into intervention
Kestenbaum et al. (2019) [40], United States	Improve pain management for patients with advance illness and unnecessary prescribing	EMR	Individual provider (w/peer comparison) and hospital level	Aggregated patient pain scores in each service region, prescribing patterns of eight medications	Monthly hand- delivered report	Palliative care clinicians	Report delivered by Chief of Medical Staff	Education session, information hand-outs, and implementation of a Preferred Drug List
Larkin et al. (2021) [45], United States	Improve ED physician Computed tomography (CT) ordering behavior	EMR (Epic)	Individual provider (w/peer comparison)	Quality indicator (i.e., CT ordering rate)	Graphical report	ED physicians	Review session with a research assistant	Education session
Navar-Boggan et al. (2014) [50], United States	Improve blood pressure control	EMR	Individual (w/peer comparison)	Quality indicator (i.e., blood pressure control, stage II hypertension)	Quarterly emailed report	Cardiologists	NR	Unspecified ongoing quality improvement initiatives
Parks et al. (2021) [49], United States	Improve adherence with intra-operative lung-protective ventilation (LPV)	EMR + anesthesia dataset	Individual provider (w/peer comparison)	Quality indicator (i.e., adherence to LPV protocol)	Interactive dashboard	Anesthetists	NR	Phased implementation: education, clinical decision support
Patel et al. (2018) [44], United States	Improve quality of discharge	EMR (Epic)	Team level	6 quality indicators (i.e., phlebotomy use, medication reconciliation)	Interactive dashboard updated daily (QlikView)	Internal medicine teams	Weekly team review of data facilitated by lead clinician	Education session
Riblet et al. (2014) [52], United States	Increase number of patients meeting the standards of care for glioma care	EMR + existing quality improvement database	Team level	10 quality indicators on peri-operative care (i.e., appropriate use of corticosteroids)	Interactive dashboard	Neuro-oncology MDTs	Quarterly team meetings led by process owners for each measure and statistician support	EMR modified to improve interdisciplinary communication, pharmacy order set, and discharge summary sent to the MDT implemented prior to intervention
Riblet et al. (2016) [51] (Phase 2 of Riblet et al. 2016)				Additional 12 quality indicators focused on acute care (i.e., post-operative complications)				New clinical pathway implemented
Trent et al. (2019) [47], United States	Improve adherence to a sepsis/pneumonia guidelines	EMR + existing quality improvement database	Individual provider (w/peer comparison) and institution level	Composite quality indicator (adherence to guidelines)	Monthly emailed report + additional emailed list of patients who received nonadherent care	ED physicians	NR	New sepsis bundle package & antibiotic implemented prior to intervention
Stevens et al. (2017) [53], United States	Reduce prescription of potentially inappropriate medications (PIMs) for older adults during ED discharge	EMR (Epic)	Individual provider (w/peer comparison)	Quality indicators (i.e., no. of patients >65 evaluated, PIM rate)	Monthly emailed report + one face to face academic detailing session	ED physicians	NR	Clinical decision support tool, pharmacy order sets, online education
Vaughan et al. (2021) [42], (Phase 2 of Stevens et al. 2017)				Quality indicators (i.e., 30-day PIM rate)	Interactive dashboard	Attending physicians and residents	Academic detailing	Education sessions led by local champions, pharmacy order sets
Wang et al. (2021) [43], United States	Improve adherence to opioid pre- scribing guidelines for the treatment of chronic non cancer-associated pain	EMR (Epic)	Individual provider (w/peer comparison) and institution level	Quality indicators (i.e., % of patients with an active opioid agreement)	Interactive dashboard (users able to create lists of patients with non-adherent care)	Rheumatologists	Initial team meeting to establish goals, action plan, divisional leadership provided coaching for prescribers who were not improving	Education session using baseline data, modified EMR to integrate local drug monitoring database/improve workflow

## Data Availability

All data relevant to the study are included in the article or uploaded as supplementary information.

## Supplementary Materials

The following supporting information can be downloaded at: https://www.mdpi.com/article/10.3390/ijerph20010200/s1, File S1: Search Strategy.
