# Supplementary material for: A Systematic Review of Electronic Medical Record Driven Quality Measurement and Feedback Systems
Source: Int J Environ Res Public Health. 2022 Dec 23;20(1):200. doi: 10.3390/ijerph20010200 (PMC9819986; doi:10.3390/ijerph20010200)
Supplement: Supplementary file 1 [file ijerph-20-00200-s001.zip › ijerph-2089010-supplementary.pdf]

Supplementary Material S1: Search Strategy

SEARCH STRATEGY: A systematic review of EMR driven quality measurement and feedback systems

Research Question: What are the characteristics and effectiveness of quality improvement interventions that utilize EMR data to drive clinical performance measurement and feedback for health professionals and clinical teams in tertiary care?

Overview: Utilising the PICO Framework, a string of search terms (example below) associated with EMR data driven performance feedback programs for health professionals and clinical teams was executed in a range of search engines. The snowball method and citation tracking was used to identify additional articles by screening the references of all included articles and using the 'cited by' feature of google scholar to screen articles that have cited the included articles since publication.

Bibliographic databases: The literature search was conducted using Medline, EMBASE, CINAHL, and Cochrane databases.

Table S1. Inclusion and Exclusion Criteria.

**Inclusion Criteria:**	**Exclusion Criteria:**
Studies within a tertiary care setting	Studies within a primary care setting
Studies that provide clinical performance feedback to healthcare professionals responsible for patient care	Studies that have a focus on non-clinical quality measures (e.g., measures on cost, workload and time management)
	Studies that provide patient information for immediate clinical
Studies that derive the measurement data from EMRs as the primary data source of analysis	decision making rather than performance feedback (i.e., clinical decision support tools)
Studies which are peer-reviewed or published in English	Studies that involve a single instance of audit and feedback
	Studies that derive the performance measurement data from time delayed clinical registries
	Studies that involve any feedback to patients
	Studies which only involved student participants (i.e., interns, residents or registrars)
	Studies which were not peer-reviewed or published in English
	Studies that only have abstracts or conference proceedings available and not full journal articles

Date range: The search incorporated results published between 1 January 2009–11 January 2022.

Table S2: Search String Terms—Example (Medline)

**Medline via Ovid**	**Concept 1**	**Concept 2**	**Concept 3**
Key concepts	Healthcare professionals & clinical teams	Performance measurement & feedback	Electronic medical record data
MeSH terms *focused MeSH term	*Patient Care Team/or exp Physicians/or *Health personnel/or *allied health personnel/or *faculty, medical/or *faculty, nursing/or *medical staff/or *nurses/or *nursing staff/or *Physician's Practice Patterns/or *Clinical Competence/	N/A	*Electronic Health Records/or *Medical Records Systems, Computerized/
Free text terms	(multi-disciplinary team OR multidisciplinary team OR MDT OR clinical team OR health* team OR healthcare team OR interdisciplinary health team OR interdisciplinary clinical team OR medical care team OR clinician OR physician OR doctor or health* practitioner OR health care practitioner OR health* professional	((audit and feedback) or performance feedback or performance monitoring or performance measurement or monitoring feedback or electronic health record feedback or performance summaries or data visuali?ation or dashboard or scorecard or scoreboard)	(computeri?d medical record* or electronic health record* or electronic patient record* or electronic medical record* or EPR or EHR or EMR or medical record* or patient record* or medical data or clinical data or patient data or practice data or hospital data)

	OR health care professional OR health* personnel OR health care personnel)
Limits applied	(English language and humans and 1 January 2009–11 January 2022)
